# Combining Real‐Time Neuroimaging With Machine Learning to Study Attention to Familiar Faces During Infancy: A Proof of Principle Study

**DOI:** 10.1111/desc.13592

**Published:** 2024-11-26

**Authors:** Elena Throm, Anna Gui, Rianne Haartsen, Pedro F. da Costa, Robert Leech, Luke Mason, Emily J. H. Jones

**Affiliations:** ^1^ Department of Psychological Science Centre for Brain and Cognitive Development, Birkbeck, University of London London UK; ^2^ Department of Psychology University of Essex Colchester UK; ^3^ Department of Neuroimaging, Institute of Psychiatry, Psychology and Neuroscience King's College London London UK

**Keywords:** EEG, infant, machine learning, Neuroadaptive Bayesian Optimisation, real‐time analysis, social attention

## Abstract

Looking at caregivers’ faces is important for early social development, and there is a concomitant increase in neural correlates of attention to familiar versus novel faces in the first 6 months. However, by 12 months of age brain responses may not differentiate between familiar and unfamiliar faces. Traditional group‐based analyses do not examine whether these ‘null’ findings stem from a true lack of preference within individual infants, or whether groups of infants show individually strong but heterogeneous preferences for familiar versus unfamiliar faces. In a preregistered proof‐of‐principle study, we applied Neuroadaptive Bayesian Optimisation (NBO) to test how individual infants’ neural responses vary across faces differing in familiarity. Sixty‐one 5–12‐month‐olds viewed faces resulting from gradually morphing a familiar (primary caregiver) into an unfamiliar face. Electroencephalography (EEG) data from fronto‐central channels were analysed in real‐time. After the presentation of each face, the Negative central (Nc) event‐related potential (ERP) amplitude was calculated. A Bayesian Optimisation algorithm iteratively selected the next stimulus until it identified the stimulus eliciting the strongest Nc for that infant. Attrition (15%) was lower than in traditional studies (22%). Although there was no group‐level Nc‐difference between familiar versus unfamiliar faces, an optimum was predicted in 85% of the children, indicating individual‐level attentional preferences. Traditional analyses based on infants’ predicted optimum confirmed NBO can identify subgroups based on brain activation. Optima were not related to age and social behaviour. NBO suggests the lack of overall familiar/unfamiliar‐face attentional preference in middle infancy is explained by heterogeneous preferences, rather than a lack of preference within individual infants.

## 1 Introduction

### 1.1 Neural Responses to Familiar and Unfamiliar Faces in Infancy

Attention allocation to social stimuli (social attention) in the first months of age is crucial for the development of the social brain (Johnson et al. 2009, Johnson 2011; Klein, Shepherd, and Platt 2009). From birth, infants are drawn to the faces of their caregivers (Sugden and Moulson 2019). Newborns can distinguish between the face of their mother and a stranger, provided they have been able to hear the mother's voice (Burnham 1993). In the first 6 months, infants continue to show preferential attention to their mother's face, potentially serving to hold their attention on an important source of comfort, nutrition and interaction. As infants grow and their social networks expand, their attention turns from their primary caregiver to new people (Jayaraman, Fausey, and Smith 2015). The pattern of the developmental change in attention to familiar and unfamiliar faces is related to social behaviour and might reflect important individual differences in social brain development (Webb et al. 2011). Furthermore, developmental changes in attention to familiar versus unfamiliar faces may reflect postnatal learning through the experience of the infant with their caregivers.

**Summary**

- Traditional group‐based experimental approaches are not always sensitive to individual differences.
- Here, Neuroadaptive Bayesian Optimisation (NBO) efficiently mapped infant neural responses across a stimulus space to identify the stimulus yielding the maximum response.
- NBO provided a lower attrition rate than traditional studies and predicted an optimal stimulus in 85% of the infants completing the study.
- Infants showed distinct attentional preferences for either the parent's or stranger's face; attentional preferences were unrelated to age and parent‐reported social behaviour.

Changing patterns of infant attention allocation to familiar and unfamiliar social stimuli have been most widely captured with the Negative central (Nc) event‐related potential (ERP) component measured with electroencephalography (EEG). The Nc is a negative deflection most prominently occurring over frontocentral electrodes between 300 and 800 ms after stimulus onset (Courchesne, Ganz, and Norcia 1981). This component is considered a neural correlate of attention engagement in the first year of age (Guy, Zieber, and Richards 2016; Richards 2003, Richards, Reynolds and Courage 2010). The Nc responds differently to faces versus objects (e.g., Conte et al. 2020; Dawson et al. 2002; Jones et al. 2016; Webb, Long, and Nelson 2005), is modulated by novelty (e.g., Carver et al. 2003; Dawson et al. 2002; de Haan and Nelson 1997, 1999; Guy et al. 2018; Guy, Zieber, and Richards 2016; Key and Stone 2012; Luyster et al. 2011; Reynolds and Richards 2005; Richards 2003; Webb, Long, and Nelson 2005), emotional expression (e.g., de Haan, Johnson and Halit 2003; Grossmann and Johnson 2007; Grossmann et al. 2011; Leppänen et al. 2007; Nelson and de Haan 1996; Stahl et al. 2010; Xie, Mallin, and Richards 2019) and familiarity (e.g., de Haan and Nelson 1997; Luyster et al. 2014; Webb et al. 2011). Thus, the Nc represents a sensitive index of the factors influencing attention allocation in infancy.

Research with the Nc has provided insight into the developmental trajectory of attention to familiar and unfamiliar faces. At 6 months of age, infants show greater Nc amplitudes to their mother's face than a stranger's face (de Haan and Nelson 1997, 1999; Webb, Long, and Nelson 2005), provided the two faces look sufficiently dissimilar (Haan and Nelson 1997). By 3–5 years, children show a reversed pattern of greater Nc amplitude towards strangers compared to the mother's face (Carver et al. 2003; Dawson et al. 2002; Moulson et al. 2009). However, the pattern of attention captured by familiar and unfamiliar faces between early infancy and childhood remains unclear. For example, one study found that at 12–17 months, typically developing children showed stronger Nc amplitudes to familiar versus unfamiliar faces (Webb et al. 2011), and a further study observed this pattern in the broader age range of 6–36 months (Luyster et al. 2014). However, other studies observed a stronger Nc amplitude for unfamiliar versus familiar faces already in the first year of age, at 12 months (Guy et al. 2018; Luyster et al. 2011) and at 9 months (Key and Stone 2012). Other studies did not observe differences in Nc amplitudes between familiar and unfamiliar faces in 12‐month‐old (Glauser et al. 2022) and 18–24‐month‐old typically developing children (Webb et al. 2011). Thus, whilst there is relatively consistent evidence for greater attention to familiar versus unfamiliar faces in young infants, and to unfamiliar versus familiar faces at 3–5 years, possibly reflecting the difference in relevance of these cues at infancy and pre‐school age, respectively, the pattern of developmental change in this effect remains unclear.

One explanation of the inconsistency in findings about the pattern of the developmental change could be related to the traditional experimental design itself. First, a key limitation to the previous literature on developmental changes in attention to faces is that typical experimental designs involve presenting the infant with two alternatives (mother and stranger), because their limited attention span precludes more options. This restricts studies to showing either a familiar or unfamiliar faces attentional preference or a null effect. This is problematic, because a null effect could reflect either *heterogeneous* individual attentional preferences, that is preferences in opposite directions between individuals that cancel out at the group level, or a *lack* of individual attentional preferences. The traditional approach does not consider the possibility that individual infants might, consistently or temporarily, be particularly engaged by faces that resemble but are not identical to their own parent's face, and show enhanced brain responses to those, possibly reflecting more processing effort for faces that are not easy to categorise into either parent and stranger. Furthermore, it is unclear how the change from attentional preference from familiar to unfamiliar faces unfolds over the second half of the first year of age—whether this is a gradual shift, or rather a sudden change, for example elicited through the learning of a new skill. New designs allowing for a larger variety of stimuli are needed to complement typical designs. Second, the traditional experimental approach requires analysis of the data after the experiment. The rich nature of EEG data generates significant analytic flexibility, which can reduce replicability (Ioannidis 2005). Additionally, when pre‐processing pipelines and analyses plans are not established a priori, systematic experimenter biases such as ‘p‐hacking’, HARKing (Hypothesising After the Results are Known), SHARKing (Selecting Hypothesised Areas after Results are known) and the use of improper statistical methods might compromise the science (Ioannidis 2005, Lorenz, Hampshire, and Leech 2017). Pre‐registration helps reduce these risks because analysis parameters are defined before data have been collected, forcing the researcher to stick to these methodological choices irrespective of the results. Although pre‐registration reduces this risk, it cannot fully solve the problem (Nosek et al. 2015.). Notably, most studies of attentional preferences to mother/stranger face have used different processing parameters, electrode selections or time windows (Table 1). Reducing the analytic variability may help increase the robustness of findings. Third, in infancy, the heterogeneity in neural processing between individuals is enhanced due to the heterogeneity of the pace of developmental change between individuals. There are substantial individual differences in infant responses that have been linked to broader social skills in some studies (though see Key and Stone 2012), suggesting that social skills may develop on different trajectories between infants, and individuals reaching milestones of social development at different rates. For example, stronger Nc amplitudes towards stranger versus parent were related to more proximity‐ and interaction‐seeking behaviours during separation and reunion with a parent at 6 months (Swingler, Sweet, and Carver 2007). Stronger Nc amplitudes towards mother's versus stranger's face were also related to increased infant distress in 6‐month‐olds (Swingler and Carver 2013). Further, stronger Nc amplitudes towards parent versus stranger were observed in 9‐month‐old infants who were quicker learners at earlier ages (Reeb‐Sutherland, Levitt, and Fox 2012), and in 12‐month‐old infants with higher expressive language scores (Glauser et al. 2022). However, these results are heterogeneous and there is little evidence of replicability. One challenge is that in traditional studies data collection is optimised for analysis at the group level, and the stability and robustness of individual‐level estimates is rarely assessed. Individualised methods that provide robust estimates at an individual level are needed to complement the traditional group‐level approach in understanding individual differences in the development of social attention.

**TABLE 1:** Summary of samples, processing choices and findings of studies examining the Nc component in response to familiar versus unfamiliar faces.

Authors	Publication year	Title	Doi	Age	Sample size	Excluded (% of total)	Stimuli	Nc feature	Nc time window	Nc channels	System	Reference	Findings
Guy, Zieber, Richards	2016	The cortical development of specialised face processing in infancy	10.1111/cdev.12543	4.5, 6, 7.5 m (cross‐sectional)	14 4.5 m, 19 6 m, 15 7.5 m TD	13 (27%)	Mother's vs. stranger's face	Peak amplitude	350–750 ms	Fz, FCz, Cz	HD system (translated into 10–10 system)	Overall average	No difference
de Haan & Nelson	1997	Recognition of the mother's face by 6‐month‐old infants: A neuro‐behavioural study	10.2307/1131845	6 m	22 TD	39 (64%)	1. Mother's vs. distinct stranger's face; 2. Two distinct stranger's face; 3. Mother's vs. similar stranger's face; 4. Two similar strangers' face	Peak amplitude, as difference between most extreme voltage in time window and the mean voltage during baseline	400–800 ms	Pz, Cz, Fz	10–20 system	Average ears	Mother > stranger
de Haan & Nelson	1999	Brain activity differentiates face and object processing in 6‐month‐old infants	10.1037//0012‐1649.35.4.1113	6 m	22 TD	22 (50%)	Mother's vs. stranger's face	Mean amplitude, peak latency	250–800 ms	Pz, Cz, Fz	10–20 system	Average ears	Mean amplitude: mother > stranger; peak latency: no difference
Nelson, Wewerka, Thomas, Tribby‐Walbridge, Odegaard Deregnier, Georgieff	2000	Neurocognitive sequelae of infants of diabetic mothers	10.1037//0735‐7044.114.5.950	6 m	34 TD, 26 IDM	26 (30%)	Mother's vs. stranger's face	Mean amplitude, peak latency	400–800 ms	Oz, Pz, Cz, Fz, T3, T4, C3, C4	10–20 system	Overall average	Mean amplitude: mother > stranger in TD only; peak latency: stranger > mother (trend)
Swingler, Sweet, Carver	2007	Relations between mother–child interaction and the neural correlates of face processing in 6‐month‐olds	10.1207/s15327078in1101_3	6 m	30 TD	25 (45%)	Mother's vs. stranger's face	Peak amplitude, peak latency	230–1230 ms	Midline: Fz, FCz; lateral: FC1, FC2, FC5, FC6, frontal: F3, F4	10–20 system	Overall average	Peak amplitude: stranger > mother in lateral electrodes
Luyster, Powell, Tager‐Flusberg, Nelson	2014	Neural measures of social attention across the first years of life: Characterising typical development and markers of autism risk	10.1016/j.dcn.2013.09.006	6, 9, 12, 18, 24, 36 m (longitudinal)	17–32 TD, 21–36 Autism infant sibs (Asibs)	NA	Mother's vs. stranger's face	Mean amplitude	390–605 ms	15, 16, 13, 9, 8, 3, 62, 61, 58, 57	HD system	Overall average	Mother > stranger in TD only (trend)
Key & Stone	2012	Processing of novel and familiar faces in infants at average and high risk for autism	10.1016/j.dcn.2011.12.003	8.5–9.5 m	20 TD, 15 Asibs	7 (20%)	Mother's vs. stranger's face	Mean amplitude, peak latency	400–600 ms	frontal: 19, 16, 10, 20, 11, 4; central: 7, 107, 32, 81, 55, REF	HD system	Overall average	Mean amplitude: stranger > mother; Peak latency: no difference
Burden, Westerlund, Armony‐Sivan, Nelson, Jacobson, Lozoff, Angelilli, Jacobson	2007	An ERP study of attention and recognition memory in infants with iron‐deficiency anaemia	10.1542/peds.2006‐2525	9, 12 m (longitudinal)	15–11 TD, 13–9 ID	24 (52%)–18 (47%)	Mother's vs. stranger's face 24 (52%)	Peak amplitude	300–650 ms	Fz, F3, F4, Cz, C3, and C4	10–20 system	Averaged mastoid	9 m: mother > stranger in IS only 12 m: stranger > mother in IS, mother > stranger in II
Luyster, Wagner, Vogel‐Farley, Tager‐Flusberg, Nelson	2011	Neural correlates of familiar and unfamiliar face processing in infants at risk for Autism Spectrum Disorders	10.1007/s10548‐011‐0176‐z	12 m	24 TD, 32 Asibs	99 (63%)	Mother's vs. stranger's face	Mean amplitude	400–850 ms	14 Frontal electrodes	HD system	Overall average	Stranger > mother
Guy, Richards, Tonnsen & Roberts	2018	Neural correlates of face processing in etiologically‐distinct 12‐month‐old infants at high‐risk of autism spectrum disorder	10.1016/j.dcn.2017.03.002	12 m	21 TD, 21 Asibs, 15 FXS	3 (0%)	Mother's vs. stranger's face	Peak amplitude	350–750 ms	Fz: 5, 10, 11, 12, 16, 18; FCz: 5, 6, 7, 12 106; Cz: 7, 31, 55, 80, 106	HD system	Overall average	Stranger > mother in TD, mother > stranger in FXS
Glauser, Wilkinson, Gabard‐Durnam, Choi, Tager‐Flusberg, Nelson	2022	Neural correlates of face processing associated with development of social communication in 12‐month infants with familial risk of autism spectrum disorder	10.1186/s11689‐021‐09413‐x	12 m	42 TD, 40 Asibs‐noA, 20 Asibs‐A	81 (44%)	Mother's vs. similarly looking stranger's face	Peak amplitude to the stranger's face subtracted from the peak amplitude to the mother's face	300–600 ms	19, 11, 4, 13, 6, 112, 7, 106	HD system	Overall average	No difference
Webb, Jones, Merkle, Venema, Greenson, Murias, Dawson	2011	Developmental change in the ERP responses to familiar faces in toddlers with autism spectrum disorders vs. typical development	10.1111/j.1467‐8624.2011.01656.x	12–30 m	15 12–17 m TD; 17 18–30 TD; 16 18–30 m autism	90 (65%)	Mother's vs. stranger's face	Mean amplitude	Early Nc: 350–550 ms, late Nc: 550–750 ms	F5 + F3: 24,25,21,30,29,36,28,35, Fz: 4,5,10,11,12,16,19,20, F4 + F6: 3,124,119,118,117,111,112	HD system	Overall average	Mother > stranger in autism and 12–17 m TD, no difference in 18–30 m TD
Carver, Dawson, Panagiotides, Meltzoff, McPartland, Gray, Munson	2003	Age‐related differences in neural correlates of face recognition during the toddler and preschool years	10.1002/dev.10078	18–54 m	14 18–24; 14 24–45; 14 45–54 m TD	33 (44%)	Mother's vs. stranger's face	Peak amplitude, peak latency	360–920 ms	Frontal electrode sites	NA	Overall average	Peak amplitude: stranger > mother; Peak latency: no difference
Moulson, Westerlund, Fox, Zeanah & Nelson	2009	The effects of early experience on face recognition: AnERP study of institutionalised children in Romania.	10.1111/j.1467‐8624.2009.01315.x	5–31 m (median age 23.5 m), 30, 42 m (longitudinal)	5–31 m: 40 TD, 81 institutionalised (IG); 30 m: 20 TD, 37 IG, 42 foster care (FCG); 42 m: 21 TD, 23 IG, 33 FCG	5–31 m: 60 (33%), 30 m: 56 (36%), 42 m: 45 (37%)	Caregiver's vs. stranger's face	Peak amplitude, peak latency	5–31 m: 350–650 ms; 30 m: 350–550 ms; 42 m: 350–550 ms	Fz, F3, F4, Cz, C3, C4	10–20 system	Average mastoid	Peak amplitude: 5–31 m: no difference; 30m: stranger > caregiver, 42 m: stranger > caregiver; Peak latency: 5–31 m: no difference, 30m: caregiver > stranger, 42 m: caregiver > stranger
Dawson, Carver, Meltzoff, Panagiotides, McPartland, Webb	2002	Neural correlates of face and object recognition in young children with autism spectrum disorder, developmental delay, and typical development	10.1111/1467‐8624.00433	34–55 m	19 TD, 16 DD, 34 autism	49 (42%)	Mother's vs. stranger's face	Peak amplitude, peak latency	194–590 ms	Left: 22, 13, 18, Ear, 11, 14, 19,12,15; central: 10, 6, 7, 8, 3, 4, 5, 54; Right: 59,1,60, Ear, 2, 61,56, 62, 57	HD system	Averaged mastoid	Peak amplitude: stranger > mother in TD only; Peak latency: no difference

Abbreviation: Asibs‐A = infant siblings of children with Autism, who also received a diagnosis of Autism, Asibs‐noA = infant siblings of children with Autism, without a diagnosis of Autism, DD: children with developmental delay, ERP = event‐related potential, FCG = foster care group, FXS = infants with fragile X syndrome, ID = iron‐deficient infants, IDM = infants of diabetic mothers, IG = institutionalised group, m = months, TD = Typically Developing children.

### 1.2 Neuroadaptive Bayesian Optimisation (NBO)

One promising methodological development that may generate significant new knowledge in this area is the advent of more sophisticated stimulus presentation and data acquisition approaches. NBO is a recently developed individualised experimental approach that aims to map the unknown underlying brain response function across one or more stimulus dimensions (Haartsen, Gui, and Jones 2024). NBO uses a closed‐loop design. It presents a stimulus selected from the range of prepared stimuli arranged along the respective dimension(s) before the session, analyses the individual's response to that stimulus, and based on this response selects the next stimulus, iteratively building up a model of the individual's response function across the stimulus space. Hence, an NBO experiment consists of two main processes: (1) alternately collecting and analysing neuroimaging data in real time, that is during the experiment, and (2) using Bayesian Optimisation (BO) to iteratively build a model of the unknown brain function based on which the next stimulus is being selected (neuroadaptive) (Lorenz et al. 2018). NBO was developed and validated in a proof‐of‐principle fMRI study with adults, involving identification of the visual and auditory properties that best evoke a target brain state (Lorenz et al. 2016). Since then, NBO has been used to address a number of questions relating to adult brain function (Lorenz et al. 2021, Lorenz et al. 2019, Lorenz et al. 2018). NBO embeds pre‐specification of experimental and analytic pipelines, fostering reproducibility of research findings (Lorenz, Hampshire, and Leech 2017), and due to its efficacy and robustness has particular value for developmental research (Gui et al. 2022).

NBO is perfectly suited to study how individual infants develop their social attention skills because it tests multiple predictions at the same time by mapping responses across a wide stimulus space, without the need to present each single stimulus. Testing multiple conditions simultaneously not only allows the testing of intermediate possibilities between stimuli and disentangling individual differences from real null effects but also allows more efficient paradigms. This is particularly beneficial for infants, who have a relatively short attention span and may become fussy, hungry or tired more quickly (Gui et al. 2022). Second, the requirement to set all analysis parameters before collecting the data carries particular value for the field of neurodevelopmental research which has yet to establish standardised analysis pipelines. Finally, the algorithm is programmed with a convergence criterion that ensures the reliability of a brain response on the individual level, only predicting an optimal stimulus when the brain response is consistent across repeated presentations. This allows individual‐level attentional preferences to be extracted more robustly than in a traditional paradigm. As a whole, NBO might be a promising method for further investigating early social development in individual infants. The technical details of the method are described below.

### 1.3 The Present Study

The present study aimed to extend the NBO approach previously applied to adult fMRI to studying infant EEG responses in the context of social development. We conducted a proof of principle study to investigate individual infants’ attention engagement with social stimuli, particularly the function of the Nc response to images of faces of their parent versus a stranger. Our first goal was to test the feasibility of this paradigm: Is it possible to achieve data from the individual infant and individual block that is reliable enough for the BO algorithm to predict an optimum before the infant becomes disengaged with the paradigm? Our second goal was identifying the individuals’ most engaging stimuli to link individual differences in attention to familiar and unfamiliar faces to age, behavioural and environmental characteristics. To this end, we collected parent‐reported information on the development of infant social behaviour through the Vineland Adaptive Behaviour Scales (VABS) and Infant Behaviour Questionnaire (IBQ). EEG was recorded in 5–12‐month‐old infants. We selected this age range because this is the period across which previous literature showed the greatest heterogeneity in findings of the group‐level Nc response towards familiar versus unfamiliar faces. We split infants into two age groups to replicate the familiarity effect previously observed in younger infants (De Haan and Nelson 1997, 1999). We hypothesised that the effect was present in this group and based our power analyses on effect sizes obtained with this age range (Luyster et al. 2014). Inconsistent previous findings did not allow a hypothesis about the effect in older infants. In other analyses, we considered age as a continuous variable.

Rather than presenting infants with a binary choice of a familiar versus an unfamiliar face, NBO allowed us to present infants with a range of face images varying in familiarity. Images resulted from morphing the caregiver's face into a stranger's face, providing greater variability in the stimulus set. Faces were ordered along a continuous stimulus space for the BO algorithm to sample across, with the parent's face and a stranger's face at its extremities. After a 12‐trial‐block of presenting one face, the Nc mean negative amplitude (“Nc mean negativity”) was calculated in real‐time and passed to the BO algorithm aiming to identify the stimulus that reliably produces the strongest Nc response in the tested infant (so‐called ‘optimum’). The experiment stopped once the NBO algorithm repeatedly selected the same stimulus for presentation or had reached a pre‐defined maximum number of blocks.

The study was preregistered on the Open Science Framework (OSF) before starting the data collection (DOI:10.17605/OSF.IO/CWF96). We hypothesised a lower attrition rate than in classic infant ERP paradigms, due to the greater variety in stimuli as well as the presentation being guided by the individual's interest. In classic paradigms, infant attrition unrelated to experimental error reaches 22% on average (23% in 5‐month‐olds and 21.3% in 10‐month‐olds; van der Velde and Junge 2020). Therefore, in the present experiment, we predicted at least 78% of the infants would complete the study. Second, we hypothesised that the distance between the parent's face and the optimal stimulus would be related to age, specifically that younger infants would show optima closer to the familiar face, in line with previous research. Third, we hypothesised that the distance between the caregiver's face and the optimal stimulus is associated with parent‐reported social behaviour and interest. Specifically, we predicted the optimum to be closer to the familiar face in infants with:

- higher socialisation scores (VABS scale),
- higher interest in familiar persons (score of selected VABS items),
- lower interest in unfamiliar persons (score of selected VABS items),
- lower distress towards unfamiliar persons (score of selected IBQ items).

To compare our individual‐level NBO results with group‐level results, we calculated the Nc amplitude in the traditional, group‐based way in response to ‛pure’ parent versus ‛pure’ stranger, ignoring the mixed images created by morphing the two originals. In line with the individual‐level predictions, we hypothesised that our target brain metric (Nc mean negativity) would be larger for the familiar compared to the unfamiliar face in the younger but not older infants, and in infants with higher socialisation scores, higher interest in familiar persons, lower interest in new persons and lower distress towards new persons. To control for a possible effect of caregiver‐stranger‐similarity, analyses were re‐run including similarity as a covariate.

## 2 Methods

### 2.1 Participants

Sixty‐one infants (*N* females = 27) aged between 5 months 0 days and 12 months 30 days (*M* = 269.10, SD = 59.40, range = 158–375) took part in the NBO experiment. For analysis, infants were split into a younger group aged 5–8 months (*N* = 29, *N* females = 13; *M* age = 217.31 days, SD = 35.37, range = 158–265), and an older group aged 9–12 months (*N* = 32, *N* females = 14; *M* age = 316.03 days, SD = 30.25, range = 272–375). Children were not invited if they had a family or personal history of epilepsy, if they were born extremely pre‐term (≤ 31 weeks of gestational age), or if they had a sensory or motor impairment or any clinical condition.

### 2.2 NBO Experiment

#### 2.2.1 Stimuli and Procedure

**FIGURE 1:**
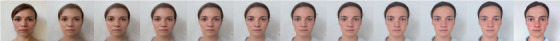
An example of the one‐dimensional parent‐stranger stimulus space.

Infants were presented with face photographs on a screen, including the face of the accompanying parent and a gender‐matched stranger. The same male or female stranger image was used across all infants. The face was centred on the image, the body below the neck was cropped and the facial expression was neutral. The image background was bright and neutral, and the faces were free from prominent accessories. The respective parent and gender‐matched stranger images were morphed into each other before each session, using StyleGAN2, a deep learning algorithm for generative image modelling (Karras, Laine, and Aila 2019), to produce 10 additional, realistic images of the respective parent's face linearly changing into stranger's face (Figure 1). The 12 images were arranged in a stimulus space, varying in the dimension of similarity to the parent's face, with the parent's face and the stranger's face as extremes of the continuum.

To account for a possible effect of similarity between the stranger's face and the respective parent's face reported previously (de Haan and Nelson 1997), after completion of the entire study two independent researchers rated the similarity of each pair of parent‐stranger faces on a continuous rating scale using a response slider ranging from 0 (very dissimilar) to 100 (very similar). Inter‐rater agreement was calculated using Pearson correlation as in De Haan and Nelson (1997). The ratings of the two independent researchers were significantly correlated (*r* [63] = 0.310, *p* = 0.012). The averaged similarity rating was included as a covariate in the analyses.

During the EEG session, the infant sat on the caregiver's lap, approximately 60 cm from a 24‐inch diagonal screen. The paradigm consisted of a maximum of 15 stimuli‐presentation blocks, with 12 trials of the same face per block. Stimuli presentation was implemented in MATLAB, using the Psychophysics Toolbox Extensions (Version 3; Brainard 1997; Pelli 1997; Kleiner, Brainard, and Pelli 2007). Each trial started with a fixation cross (500–1000 ms), followed by the face image on a grey background for 500 ms. After the end of a block, that is after the 12^th^ trial, a colourful still image was presented on the screen, whilst the EEG data obtained during the block was analysed and the optimisation was performed (∼ 6 s). Procedures to monitor and attract the infant's attention to the screen are described in Supporting Information 1.

#### 2.2.2 Data Acquisition

EEG data were recorded using the gel‐based, wireless ENOBIO 8‐channel EEG system (NE Neuroelectrics; 10‐10 EEG coordinate system) with 6 fronto‐central electrodes of interest (Fz, FC1, FC2, C1, C2 and Cz) and two reference electrodes (P7 and P8). The system's two default electrodes for online referencing (common mode sense, CMS; driven right leg, DRL) were attached to the right mastoid behind the ear. EEG data was digitised at 500 Hz. Before the start of the experiment, the researcher ensured that the NIC2 quality index including noise and offset of the signal was orange or green in the eight electrodes and that the EEG signal looked good (e.g., not noisy) by visual inspection. Lab streaming layer (LSL) was used to stream the EEG data and read it into MATLAB during the experiment.

#### 2.2.3 EEG Pre‐Processing and EEG Target Metric Calculation

After each block, the streamed EEG signal was pre‐processed using custom MATLAB scripts. The raw EEG data was cut into 1500 ms segments around the stimulus marker. Segments were detrended, demeaned, mirror‐padded (padding value: 1000) and band‐pass filtered (0.1–20 Hz). Mirror‐padded segments were cut around stimulus onset (100 ms before to 800 ms after) and baseline‐corrected. For each channel, artefactual trials were excluded if the signal exceeded an individually defined amplitude (i.e., 250 or 200 µV) and/or range threshold or was consistently flat (Supporting Information 2). The signal was averaged across all clean trials and channels of interest (Fz, Cz, FC1, C1, FC2 and C2). The pooled signal of the reference channels P7 and P8 was subtracted from the pooled signal of the channels of interest. Finally, the Nc mean negativity was calculated as the mean amplitude of the biggest negative deflection within the broad Nc time window of 250–800 ms (de Haan and Nelson 1999; Supporting Information 3).

#### 2.2.4 Real‐Time EEG Data Quality Check

**FIGURE 2:**
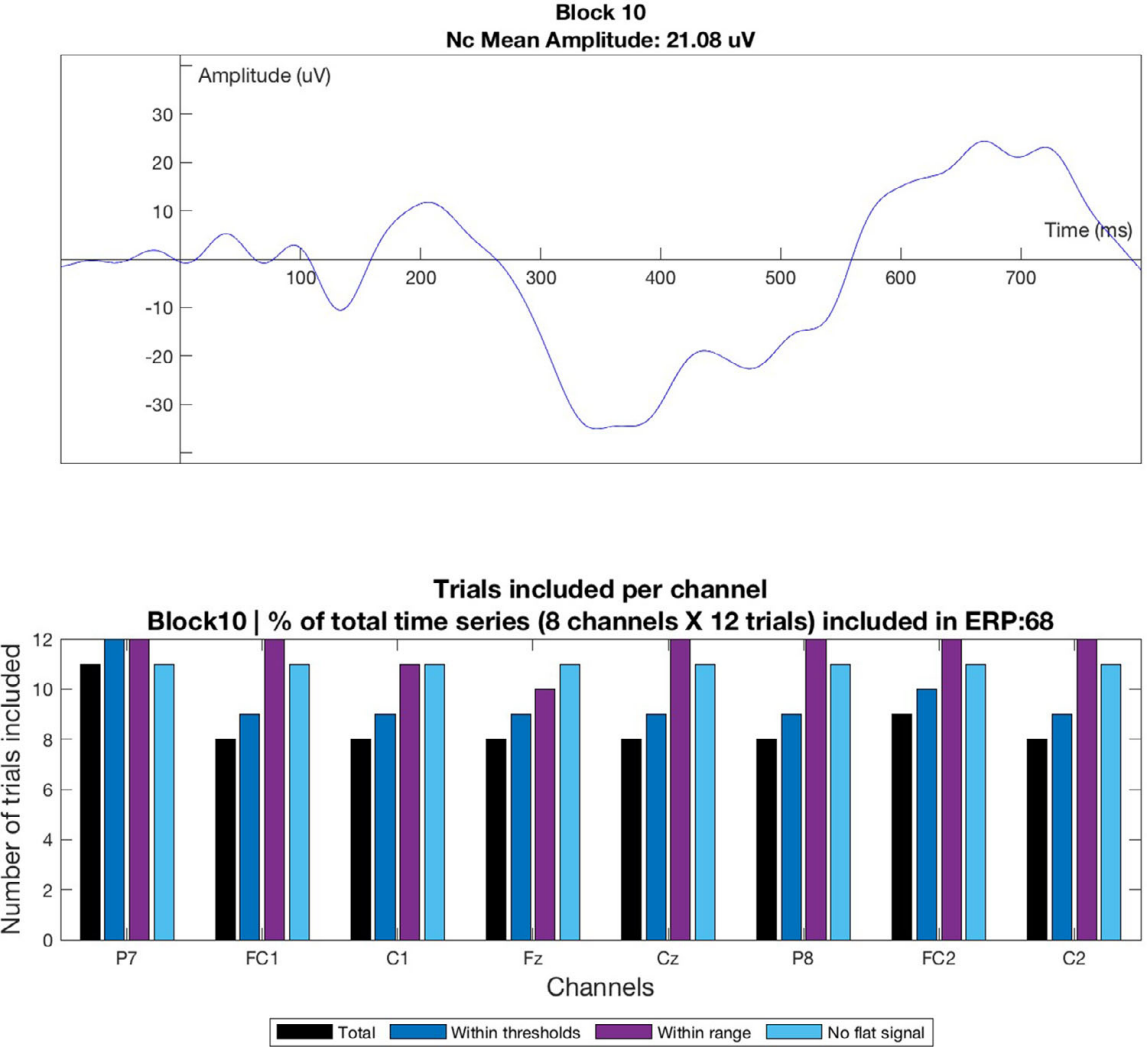
Bar chart displaying the Nc (top) and number of valid trials per channel (bottom) for the current block.

Before passing on the EEG target metric to the BO algorithm, further automated and manual real‐time EEG data quality checks were performed in MATLAB. First, the percentage of trials that survived artifact rejection from all recorded trials across the channels of interest (6 × 12 trials = 72 trials) was calculated. In order for the EEG target metric to be passed to the BO, it had to include at least 10 artifact‐free trials, in line with previous Nc research (Gui et al. 2021; Burden et al. 2007; Luyster et al. 2011, Moulson et al. 2009; Key and Stone 2012; Glauser et al. 2022). If this threshold was not met, the EEG target metric was not passed on to the BO, and instead the entire block was repeated (maximum 2 repetitions allowed before paradigm stopped automatically due to poor data quality). As an additional real‐time data quality check, the number of valid trials per channel was plotted after each block (Figure 2). This allowed the researcher to identify potential channels of poor quality, giving the opportunity to undertake adjustments on the cap to improve the signal quality.

#### 2.2.5 NBO

NBO combines real‐time analysis of neurophysiological or neuroimaging data with artificial intelligence to identify from a range of stimuli the one maximising a target brain state in an individual participant (da Costa et al. 2021; Lorenz et al. 2016). In a closed‐loop experimental design, a surrogate model of the unknown function of the participant's brain response is mapped across the stimuli arranged in a meaningful space. In each iteration of the loop, the surrogate model is iteratively updated by empirical data recorded in response to the respective stimulus presented, until the model predicts extrema. This way, model extrema are rapidly identified whilst only a subset of the stimuli are presented. A pre‐defined acquisition function selects the stimulus to present next, based on features of the surrogate model, and defines the degree to which the BO algorithm favours exploring uncertain stimuli versus exploiting stimuli where it predicts maxima.

In this study, the BO algorithm was programmed towards maximising the Nc mean negativity by sampling towards the optimal point in the parent‐stranger stimulus space. Of note, we aimed to elicit the most *negative* Nc mean amplitude value, representing a stronger Nc and hence higher attentional engagement with the stimulus. An initial set of four pre‐selected stimuli were presented as ‛burn‐ins’ to provide the algorithm with an initial model to start the optimisation from. These four initially sampled images corresponded to the two extreme points of the stimulus space (100% parent, 100% stranger) as well as two points from the middle of the search space (33% stranger/66% parent, 66% stranger/33% parent). The order in which these four stimuli were presented was randomised across participants, to prevent potential confounding effects of the presentation order on the neural measure. The two images of parent and stranger were consistently included in the set of burn‐ins to provide the BO with the maximum variety of different points across the space to predict an initial model of the neural response. The early stopping criterion of convergence, that is, of when the optimal stimulus can be considered identified, is set by the researcher prior to the study (Lorenz et al. 2015), and was here defined to be achieved if the same stimulus was sampled in three consecutive iterations (as in Lorenz et al. 2018), which results from the BO consistently predicting a specific face image to elicit the maximum Nc mean negativity. Reaching this stopping criterion is only possible if the responses mapped on the stimulus space are reliable, with stronger signals consistently being concentrated in the same region of the stimulus space of an individual. If this stopping criterion indicating convergence was not reached, the paradigm stopped automatically after 15 blocks, to avoid exceeding the infant attention span. A more detailed description of the BO‐algorithm used in the present study can be found in the Supporting Information 4 and in da Costa et al. (2021). The entire pipeline, including EEG testing Standard Operating Procedures and scripts, are available online: https://osf.io/yfa6t/.

### 2.3 Parent‐Report Measures of Social Development

Parents were asked to fill in online questionnaires about their child's social behaviour and interests prior to their visit to the lab. This study included the VABS—II edition (Sparrow, Cicchetti, and Balla 2005) and the Infant Behaviour Questionnaire‐Revised (IBQ‐R; Gartstein and Rothbart 2003). Please see Supporting Information 5 for a description of these questionnaires. Preregistered analyses included the variables ‘Interest in a familiar person’ and ‘Interest in a new person’, which are indices resulting from the combining of selected items of the VABS, and the variable ‘Distress towards other persons’, which is an index resulting from combining selected items of the IBQ (see Supporting Information 6 for the selected items). For each infant, the mean across the raw scores of the items contributing to each of the three indices was calculated and used as a variable in the present study.

### 2.4 Statistical Analysis After Data Collection

#### 2.4.1 Attrition Rate

Our measure of attrition was the proportion of infants completing the experiment, that is, infants who either reached the early stopping criterion or the maximum of 15 blocks. To achieve a measure of convergence, that is for identification of the optimum, we calculated among these infants who completed the experiment the proportion of infants who reached the early stopping criterion. The following statistical analyses of the individual optima only included the infants for whom the BO had converged.

#### 2.4.2 Optimum‐Parent Distance

The primary outcome of the experiment was the position of the individual optimum in the parent‐stranger stimulus space obtained after each infant's session. The position of the individual optimum was operationalised as Euclidean distance from the parental face (‛optimum‐parent distance’, continuous), with a shorter optimum‐parent distance reflecting an optimal stimulus closer to the parent's face in the parent‐stranger stimulus space (i.e., more similar to parent).

#### 2.4.3 Convergence Towards Parent Versus Stranger Face

The proportion of optima in the parent and the stranger half of the stimulus space across the entire sample was calculated, respectively.

#### 2.4.4 Relation to Age

An ANOVA was used to test whether the optimum‐parent distance differed by age group (5–8 m versus 9–12 m). A simple linear regression was used to test whether the optimum‐parent distance was predicted by age in days.

#### 2.4.5 Relation to Behaviour

Multiple linear regression was used to test whether the optimum‐parent distance was predicted by the ‘Interest in familiar persons’ score, the ‘Interest in other persons’ score and the ‘Distress towards other persons’ score. Age in days was included as a covariate in the model.

#### 2.4.6 Additional Logistic Regressions

Given that the optima were observed to be clustered at either the parent's or stranger's face in the stimulus space, instead of being spread across the continuous parent‐stranger stimulus space as anticipated, we ran additional, non‐preregistered logistic regressions to test whether the likelihood of converging at either the parent‐ or the stranger‐side of the stimulus space was associated to age or behaviour.

#### 2.4.7 Parent‐Stranger Similarity

In exploratory linear regressions, we additionally included rated parent‐stranger‐similarity as a covariate to see whether it related to the optimum parent distance.

#### 2.4.8 Group‐Level Results

We also tested how far the group‐level results from the present experiment matched results from previous group studies (preregistered), and in additional non‐preregistered analyses how far the individual NBO results matched the group‐level results from the present experiment.

To test how far the present group‐level NBO results match results from previous studies, we calculated the Nc mean amplitude across the broad 250–800 ms time window, to the 100%‐parent‐ and 100%‐stranger‐images presented. Repeated‐measures ANOVA with all participants who provided data for the parent and stranger block during the burn‐in phase (*N* = 57, preregistered) was used to test whether, at a group level, the Nc mean amplitude differed by stimulus condition (parent's vs. stranger's face) and/or age group (5–8 m vs. 9–12 m). Based on previous studies, we predicted a significantly stronger Nc mean amplitude for parent versus stranger's face in the group of the 5–8‐month‐olds only.

To test how far the individual NBO results matched the group‐level results from the present experiment, we calculated for each infant the Nc mean negativity towards all 100%‐parent and ‐stranger images presented across the experiment. Repeated‐measures ANOVA with all infants who had reached convergence and provided individual BO optima (*N* = 44, non‐preregistered) was used to test whether, at a group level, the Nc mean negativity differed by stimulus condition (parent's vs. stranger's face) and/or age group (5–8 m vs. 9–12 m). In additional non‐preregistered analyses, we compared the average Nc mean negativity to 100%‐parent versus 100%‐stranger photo in two sub‐groups of infants separately: in infants for whom the BO had converged at the parent side of the stimulus space (“familiar optimum” subgroup), and at the stranger side (“unfamiliar optimum” subgroup), respectively, to test whether individual optima could identify subgroups of infants.

## 3 Results

### 3.1 Overall Attrition and Convergence

**FIGURE 3:**
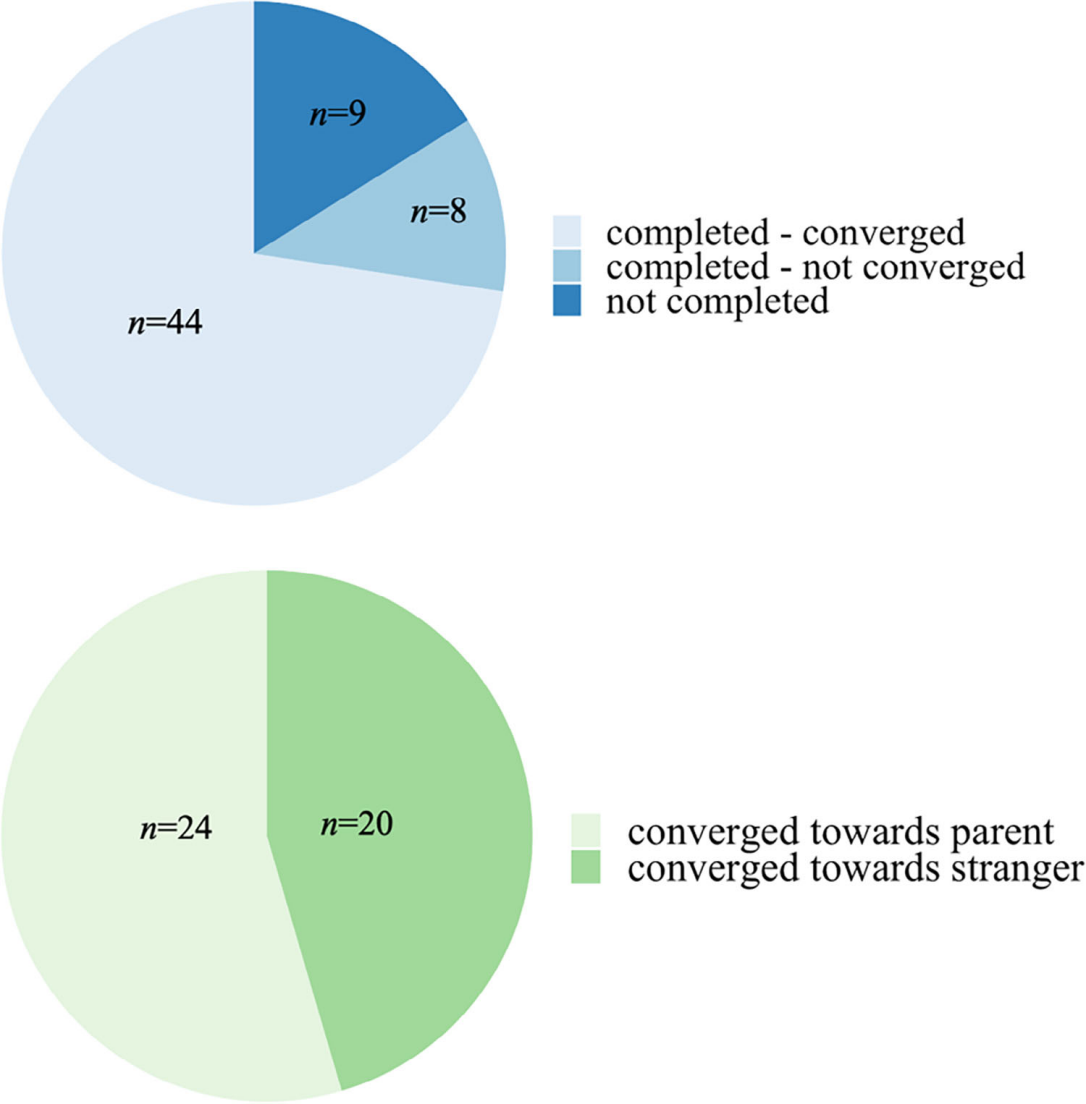
Top: Proportion of infants who completed the paradigm and converged or did not converge within 15 blocks. Bottom: Proportion of infants who converged on the parent and stranger side of the stimulus space, respectively.

The results of the different measures are listed in Table 2. Of the 61 infants who participated, 52 infants completed the experiment (85.25%). The remaining infants did not provide enough good‐quality data, and/or the experiment had to be interrupted due to fussiness. Although we preregistered to end data collection only when reaching a sample size of 60 infants with good quality data for the statistical analysis, data collection was interrupted prematurely due to measures to prevent the spread of COVID‐19. Of the 52 infants who completed the study, 44 infants reached the early stopping criterion indicating convergence (84.62%; *N*_5‐8 mo_ = 19, *N*_9‐12 mo_ = 25), whereas the remaining 8 infants (15.38%) completed the maximum number of blocks without converging to an optimum (Figure 3). The mean number of blocks presented before convergence was 10.34 (SD = 2.26, range: 6–14; Table 2). Whether the NBO converged did not depend on experimenter‐rated parent‐stranger similarity (*β* = −0.001, SE = 0.025, *p* = 0.984).

**TABLE 2:** Different measures of the infants who reached the early stopping criterion (“convergence”) and hence were entered in group analysis (*n* = 44).

Mean (standard deviation, range)			
	Total (*n* = 44)	6–9 m (*n* = 19)	9–12 m (*n* = 25)
**Age in days**	277.20 (55.96,161–375)	225.68 (34.60, 161–265)	316.36 (32.13, 272–375)
**Parent‐report measures**			
Vineland socialisation	90.51 (13.96, 49–123)	91.88 (18.52, 49 123)	89.37 (8.90, 70–103)
Interest in familiar person	1.81 (0.27, 1–2)	1.74 (0.33, 1–2)	1.87 (0.19, 1.33–2)
Interest in unfamiliar person	1.78 (0.29, 1–2)	1.67 (0.34, 1–2)	1.87 (0.22, 1.33–2)
Distress towards unfamiliar person	1.63 (1.33, 0–4.33)	0.93 (0.89, 0–2.67)	2.17 (1.37, 0–4.33)
**EEG measures**			
Nc mean negativity familiar face (µV)	14.08 (7.74, 0.35–39.71)	12.92 (10.05, 0.35–39.71)	14.95 (5.47, 6.68–30.78)
Nc mean negativity unfamiliar face (µV)	15.03 (7.32, 2.68–33.72)	15.14 (8.74, 3.37–33.72)	14.95 (6.21, 2.68–27.41)
**NBO output measures**			
Efficiency (number of blocks for convergence)	10.34 (2.26, 6–14)	10.79 (1.96, 8–14)	10.00 (2.45, 6–14)
Euclidean distance between optimum and familiar face	1.00 (1.04, 0–2.2)	1.08 (1.05, 0–2.2)	0.94 (1.05, 0–2.2)

### 3.2 Overall Attentional Preference for Familiar versus Unfamiliar Faces

Of the 44 infants who converged, that is, for whom the optimum was considered identified, 20 (45.45%) infants converged for the 100%‐parent's face, 16 (36.35%) converged for the 100%‐stranger's face and 8 infants (18.20%) converged for one of the morphed faces between the two extremes. Splitting the stimulus space into a parent half and a stranger half, 24 infants (54.55%) converged on the parent side of the stimulus space, whilst 20 infants (45.45%) converged on the stranger side (Figure 3). A 2‐sample test for equality of proportions indicated that this difference was not significant (*X* ^2^[1, 2] = 0.409, *p* = 0.522).

### 3.3 Relation to Age

In the 44 infants who converged, the *probability of convergence* was not predicted by age in days (*β* = 0.002, SE = 0.007, *p* = 0.721). The *optimum‐parent Euclidean distance* did not differ significantly between age groups (*F*[1,43] = 0.273, *p* = 0.604) and was not predicted by age in days (*β* = −0.004, SE = 0.002, *p* = 0.115). Including rated parent‐stranger similarity as a covariate did not change the pattern of results (*p*s > 0.1 in all models). Non‐preregistered logistic regressions showed that the likelihood of converging at either the parent‐ or the stranger‐side of the stimulus space was not significantly associated with age (see Supporting Information 7).

### 3.4 Relation to Behaviour

In the 44 infants who converged, the *probability of convergence* was not related to infants’ interest in familiar persons (*β* = −1.689, SE = 2.204, *p* = 0.443), interest in unfamiliar persons (*β* = −0.930, SE = 1.854, *p* = 0.616) and distress towards unfamiliar persons (*β* = −0.136, SE = 0.318, *p* = 0.669).

In the 44 infants who converged, the *optimum‐parent Euclidean distance* was not significantly predicted by interest in familiar persons (*β* = 0.453, SE = 0.709, *p* = 0.527), and interest in unfamiliar persons (*β* = −0.265, SE = 0.654, *p* = 0.687), and distress towards unfamiliar persons (*β* = 0.132, SE = 0.152, *p* = 0.392). Adding in rated parent‐stranger similarity did not change the pattern of results (all *p*s > 0.2). Non‐preregistered logistic regressions showed that the likelihood of converging at either the parent‐ or the stranger‐side of the stimulus space was not significantly associated with behaviour (see Supporting Information 7). Further, the likelihood of converging at one of the extremes versus the middle of the space was not significantly predicted by interest in familiar persons (*β* = 0.453, SE = 0.709, *p* = 0.527), interest in unfamiliar persons (*β* = −0.265, SE = 0.654, *p* = 0.689) or distress towards unfamiliar persons (*β* = 0.131, SE = 0.152, *p* = 0.392).

### 3.5 Group‐Level Nc Amplitude Analysis

**FIGURE 4:**
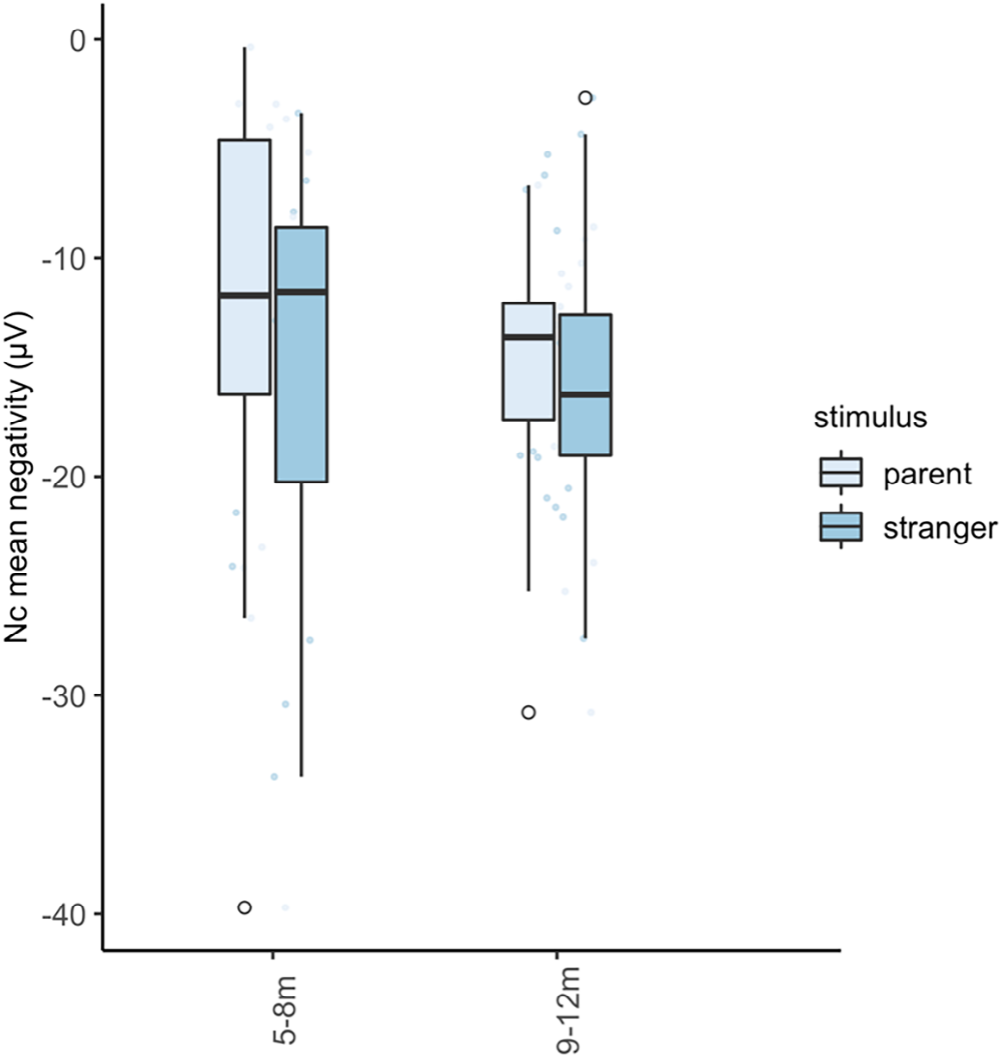
Nc mean negativity in microvolt (EEG target metric for the NBO‐algorithm), by (original 100%) parent versus stranger stimulus and by age group.

To compare individual‐level with group‐level results of the present study, we calculated the Nc mean negativity towards all original photos of 100% parent and 100% stranger presented during the experiment. In the 44 infants who converged, repeated‐measures ANOVA showed that the Nc mean negativity towards all original photos of 100% parent versus 100% stranger did not significantly differ between conditions (*F*[1,42] = 0.63, *p* = 0.43, *η*_p_^2^ = 0.01), age group (*F*[1,42] = 0.22, *p* = 0.64, *η*_p_^2^ = 0.01) and their interaction (*F*[1,42] = 0.83, *p* = 0.37, *η*_p_^2^ = 0.02). Adding in rated parent‐stranger similarity did not change the pattern of results; also, similarity itself did not have a significant effect on the Nc (*F*[1,41] = 0.09, *p* = 0.77, *η*_p_^2^ = 0.002). Means are visualised in Figure 4.

To obtain a measure that is comparable with the previous literature, we calculated the Nc mean amplitude by averaging the amplitude across the entire 250–800 ms time window rather than only for the period of negative deflection, towards the parent and stranger block of the burn‐in phase. Consistent with the analysis of the Nc negativity, this repeated‐measures ANOVA revealed no significant effect of condition (*n* = 57, *F*[1,55] = 0.153, *p* = 0.697), age group (*F*[1,55] = 0.221, *p* = 0.640) and their interaction (*F*[1,55] = 0.034, *p* = 0.853). Adding in rated parent‐stranger similarity did not change the pattern of results (all *p*s > 0.6). The grand average Nc waveform is visualised in Supporting Information 8.

**FIGURE 5:**
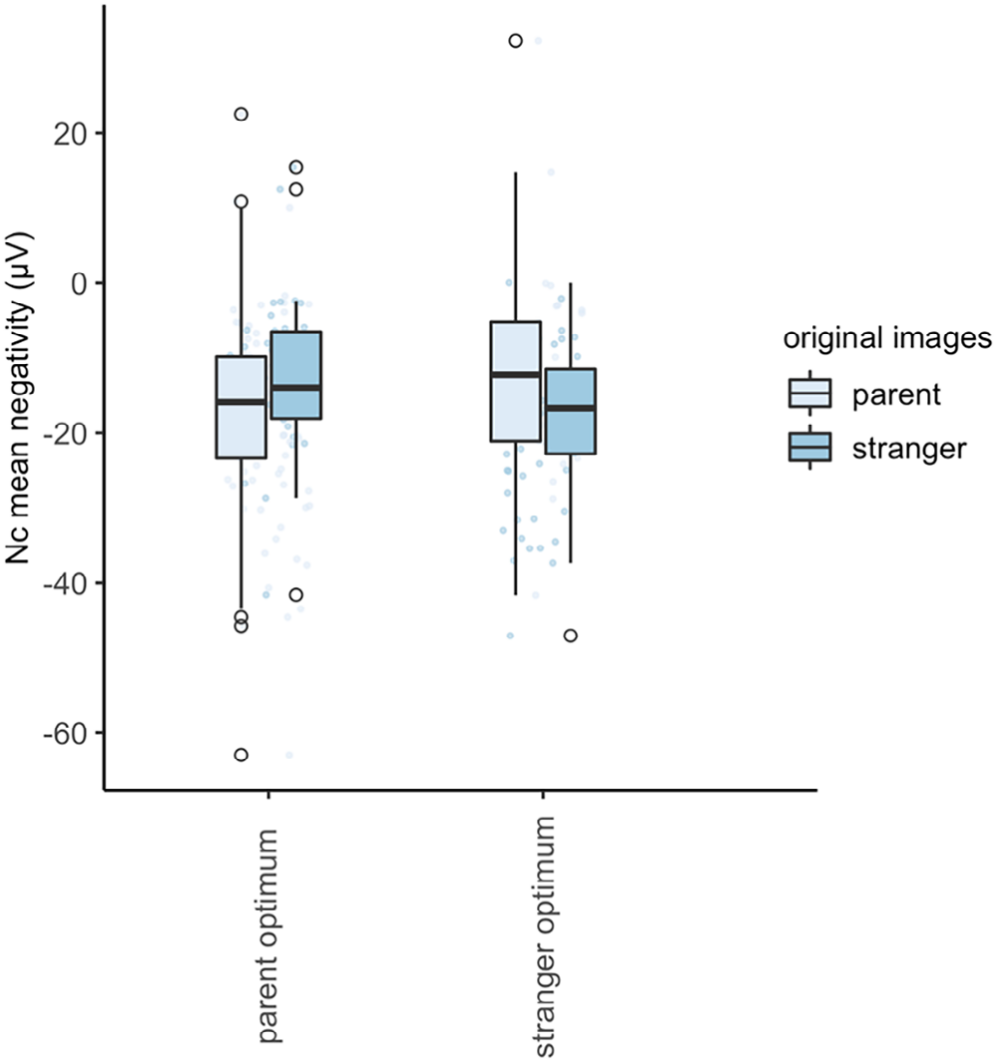
Nc mean negativity towards original parent and stranger images in the parent optimum subsample and stranger optimum subsample.

We then split the group into subgroups of infants who had converged at the parent side (‛familiar optimum’ subgroup, *n* = 24) and the stranger side (‛unfamiliar optimum’ subgroup, *n* = 20) of the stimulus space, respectively. Re‐running the analysis in each subgroup revealed a significantly stronger Nc mean negativity for parent versus stranger in the ‛familiar optimum’ infants (*F*[1,23] = 15.39, *p* < 0.001, *η*_p_^2^ = 0.40), and a significantly stronger Nc amplitude for stranger versus parent in the ‛unfamiliar optimum’ infants (*F*[1,19] = 30.39, *p* < 0.001, *η*_p_^2^ = 0.62; Figure 5).

Additional control analyses and visualisation of the Nc mean negativity as a function of block number indicate that results were not affected by the number of blocks (Supporting Information 9).

## 4 Discussion

### 4.1 Summary

Social brain development in early childhood is shaped by attention allocation to social stimuli. With increasing experience with their caregivers, infants show developmental changes in their attention to familiar versus unfamiliar faces, and these changes relate to social behaviour skills in toddlerhood. However, group studies reported inconsistent findings regarding the pattern of this change in infant attention after the first 6 months of age. This might be because this attentional preference differs between individuals, because infants do not have an attentional preference at that age range, or because infants prefer intermediate faces (between familiar and unfamiliar) at that age. In this study, we tested the utility of a novel method, NBO, to study individual infants’ attention engagement with faces varying in the degree of similarity to their parent's (familiar) and a stranger's (unfamiliar) face.

With 85% of the infants completing the study, attrition rate (15%) was lower than in many traditional infant ERP studies, confirming the practical utility of the method. The BO converged to an optimum in 85% of the infants completing the study (72% of all infants tested). The proportion of individual optima did not significantly differ between the parent versus the stranger side of the stimulus space in infants aged between 5 and 12 months; the position of the individual optimum was not related to age or measures of social behaviour. However, group analyses confirmed significantly stronger responses to the familiar than unfamiliar face and unfamiliar than familiar face when sub‐grouped into infants for whom the algorithm converged at the familiar versus unfamiliar face respectively. This provides further confirmation that the individual‐level attentional preferences identified by the algorithm were robust and indicates that similar group‐level responses to familiar and unfamiliar faces in middle infancy relate to heterogeneity in attentional preference, rather than an intermediate attentional preference or no attentional preference. However, whilst individual infants do show robust attentional preferences for particular faces, these were not systematically related to age or social behaviour in this developmental window.

### 4.2 Feasibility and Practical Advantages of NBO

The attrition rate of the present study (15%) was lower than has been previously reported in infant EEG studies using classic designs (21%–23%; van der Velde and Junge 2020) and in previous reports looking at the Nc in response to familiar versus unfamiliar faces (Table 1). Reduced attrition due to infant fussiness or tiredness could be related to the infant‐guided stimulus presentation and larger stimulus variety embedded in the NBO study design through increased task commitment (Gui et al. 2022).

For 85% of the infants completing the study, the NBO converged within on average 10 blocks, suggesting the Nc negativity value fed to the algorithm was a sufficiently reliable measure of the underlying brain process under test (attentional engagement to faces) and allowed to rapidly reduce the uncertainty in the predicted response function modelling the relationship between the search space and the Nc. This finding also indicates that reliable data can be achieved with low‐density EEG including only eight electrodes as used in the present study. The EEG system was selected to be low‐density to ensure a quick cap preparation via pre‐gelling and to minimise the weight on the head of the infants. It was further selected to be wireless to enable infants to move freely in the stimulus presentation breaks and provide a proof‐of‐principle for more naturalistic future paradigms. Of note, many historic studies focusing on the Nc component recorded it from single electrodes (e.g., Richards 2003). We have previously recorded reliable ERP data with this system in toddlers (Haartsen et al. 2021). That the algorithm converged to optima for most infants demonstrates sufficient reliability in the recorded responses despite the relatively low number of electrodes. The experiment stopped automatically if data quality did not reach pre‐defined thresholds for an individual. These observations may have particular utility for research in large‐scale studies that are seeking biomarkers on an individual level. Non‐convergence in the remaining infants (15%) in the present study may reflect either unreliability of the target metric in these infants or lack of attentional preference for parent versus stranger.

### 4.3 Familiar and Unfamiliar Face Processing

In our sample of 44 infants aged between 5 and 12 months, we did not observe an attentional preference for the familiar or the unfamiliar faces as measured by the group‐level Nc ERP. The age‐group finding adds to the familiar/unfamiliar literature, contrasting previous findings of stronger Nc amplitudes towards mother versus stranger both at 6 months (de Haan and Nelson 1997, 1999, Nelson et al. 2000), 9 months (Burden et al. 2007) and at 6–36 months (Luyster et al. 2014). Although a trend towards stronger Nc amplitudes for unfamiliar faces was observed in our data, this was non‐significant, differently from what was reported for infants aged 6 months (Swingler, Sweet, and Carver 2007), 9 months (Key and Stone 2012) and 12 months (Guy et al. 2018; Luyster et al. 2011, Burden et al. 2007). The present findings are consistent with studies reporting no difference in Nc amplitude between parent's and stranger's face in 12‐month‐old infants (with a similar trend towards greater amplitude in typically developing infants; Glauser et al. 2022) and are further confirmation of the heterogeneity present in these responses. Our analyses were planned to be sufficiently powered to detect previously reported effects within the age groups, with a similar sample size per age group as the above‐mentioned studies (*N*_5‐8 mo_ = 22, *N*_9‐12 mo_ = 32 in Luyster et al. 2014, versus *N*_5‐8 mo_ = 19, *N*_9‐12 mo_ = 25 in the current sample; see Table 1). Additionally, as discussed, data were only included in the analyses if they passed multiple layers of quality checks. These null findings are therefore important as they raise questions about evidence from previous traditional approaches to studying the complexity of social behaviour (Almaatouq et al. 2022). The absence of clear group results should also provide further motivation towards moving away from approaches that study development by averaging responses to capture a ‘normative’ pattern, to individualised approaches that investigate why and how infants differ in their developmental paths.

Interestingly, 80% of the infants converged on one of the extremes of the stimulus space (i.e., 100% parent or 100% stranger's face), whilst only 20% converged to a point in between. Of note, the fact that in most cases the algorithm converged over the original parent or stranger photo does not stem from these images being included in the set of burn‐ins, because the Nc mean negativity was not generally higher at the beginning of the experiment and subsequently decreasing (Supporting Information 8), and further, the other two burn‐in images were not frequently selected as optima. This pattern of results indicates that the infants’ Nc was indeed sensitive to the difference between the two faces and indicative of attentional preferences; these were just heterogenous within the group. This can be contrasted with a situation in which infants may all fail to converge, which would indicate a lack of a differential response across the space. For example, Dawson et al. (2002) showed that 3–4‐year‐old toddlers with autism did not show the typical amplitude difference in the Nc (and two other ERP components) towards familiar versus unfamiliar faces, interpreted as suggesting that the brain of autistic toddlers did not discriminate between their caregiver and a stranger. Further, typically developing children showed the absence of an attentional preference on the group level pattern at an earlier age, before showing an attentional preference for unfamiliar face on the group level, suggesting that in autism this shift is delayed (Webb et al. 2011). In light of the present findings, one could speculate that the non‐significant difference in attentional preference to familiar versus unfamiliar faces in autistic toddlers (and in typical individuals at an earlier stage) resulting from group‐level analyses may not reflect an absence of an attentional preference in these individuals, but rather that individual attentional preferences at that stage might be heterogeneous. To support this idea, it would be important to apply NBO to study individual attentional preferences to familiar versus unfamiliar faces in autistic toddlers.

These findings illustrate the utility of the NBO approach in distinguishing between heterogeneity in responses at the level of the individual infant, and a common lack of attentional preference for either stimulus. The cognitive interpretations of these two patterns are very different (individual differences in attentional preference but ability to discriminate versus lack of attentional preference or discrimination). However, contrary to preregistered predictions, we did not observe a change in the preference towards familiar or unfamiliar faces across ages over the second half of the first year of age. Visual inspection of the Nc values suggested that a tendency towards an attentional preference for stranger versus parent was present in both age groups (Figure 4). Since we only focused on an age range within the first year of age, it is possible that an age‐related shift in this direction may happen later in toddlerhood or early childhood. Alternatively, the observed trend of attentional preference for unfamiliar over familiar faces between 5 and 12 months, without a significant difference between younger and older infants, could stem from the fact that the younger age group in the present study (including children ranging from the fifth to the end of the eighth month) was, on average, older than the previously studied samples of 6‐month‐old infants showing a stronger effect for parent versus stranger (de Haan and Nelson 1997, 1999). Individual differences in attentional preferences for familiar/unfamiliar faces at this age may relate to other characteristics of the infant's family or home life that we did not measure in this study.

### 4.4 Relation Between Preferred Stimulus and Social Behaviour

We had predicted that individual optima closer to the parent's face would be related to behavioural measures of individual differences in interest in familiar and unfamiliar faces and distress when seeing unfamiliar persons. Our results show that the choice of face images that produces the strongest attentional engagement in the infant's brain is not directly related to parent‐reported overt behaviour in relation to people. Observable signs of the child's behaviour in naturalistic social situations, for example during parent‐child interaction, might be better able than parent report measures to explain differences in Nc amplitude towards familiar and unfamiliar faces in the first year of age. For example, at 6 months, smaller Nc amplitudes towards the parent and stronger Nc amplitudes towards a stranger's face were related to increased looking for the parent during separation (Swingler, Sweet, and Carver 2007), whilst greater Nc amplitudes towards the parent were related to increased infant distress during naturalistic interaction (Swingler and Carver 2013). A wider investigation of behavioural traits that could be linked to the NBO results would be worthwhile to take a step further in understanding differences in developmental trajectories. However, here we focused on the preregistered approach to avoid any risk of false positives, given the novelty of the paradigm. The present study generates new hypotheses and ideas for future investigations, which can be preregistered ahead of time. One interesting avenue of research concerns relating the NBO results to the infants’ social environment. Particularly, it would be interesting to test the extent to which individual attentional preferences along a continuum between familiar versus unfamiliar faces are linked to how much the infants were exposed to new faces in everyday life. This dataset will be particularly interesting in the context of the COVID‐19 pandemic, since all infants who took part in the present study were born in the year 2021 or early in 2022, a time on a societal level characterised by more social distancing, less participation in social events, and faces outside the household covered by masks. Since early exposure to the entire face or to its internal features plays a role in the development of face processing skills and social cognition in general (Carnevali et al. 2022), it would be interesting to see whether there was a relation between the number of new faces the child was exposed to in everyday life and their attentional preference for familiar versus unfamiliar faces.

### 4.5 Limitations and Future Directions

We had to apply some changes compared to the original preregistered plan. First, we had aimed for a target sample size of *N* = 30 infants for whom the BO converged per age group. Due to delays, laboratory disruptions and forced procedural changes imposed by the COVID‐19 pandemic, we only collected data of *N* = 19 younger and *N* = 25 older infants for whom the BO converged. The previous target sample size was calculated in a G*Power analysis, revealing that based on Luyster et al. (2014) and a power of 80%, we would need *n* = 29 6‐month‐old infants to obtain a significantly greater response for a familiar versus unfamiliar face. In the present sample, the effect was not significant (*F*[1,18] = 0.916, *p* = 0.351, *η_p_^2^* = 0.05, Figure 4), and the Nc amplitude was stronger for the stranger's (*M* = 15.14) versus parent's (*M* = 12.92) face. Given this size of the effect (*η_p_^2^* = 0.05, Cohen's *f* = 0.22), we would need a sample size of 152 infants for it to be significant. Second, in our pre‐registration, we included analyses on the total VABS Socialisation Scores that had to be excluded from the current report due to a mistake in the study set‐up. Thus, this analysis is not reported here, since the VABS Interpersonal Relationships subdomain that contributes to this score was accidentally not fully administered, resulting in the ceiling level not being established in many infants.

We also performed additional analyses that were not preregistered. One additional analysis included subgrouping by parent‐ or stranger‐optimum to test whether the Nc mean negativity pattern was consistent with the subsample group (greater for parent in the ‛familiar optimum’ subgroup, greater for stranger in the ‛unfamiliar optimum’ subgroup). Although this result cannot be considered an independent verification of the NBO results, as the subsample data were not independent from the overall sample, it confirms that the optimisation did not just centre on a random fluctuation but that the individual optimum is indeed reflecting this infant's Nc response when analysed on the group‐level. Our pre‐registered approach using the Nc mean negativity aimed to capture a personalised signal reflecting the Nc amplitude and latency; we acknowledge that different operationalisations could be tried in future studies, for example defining the most negative peak within the time window and calculating the mean amplitude of the ERP around it.

In this proof‐of‐principle of the feasibility of the NBO we used a relatively simple, one‐dimensional space. Therefore, the current paradigm did not allow us to fully exploit the potential of the NBO approach to present virtually infinite stimuli and test multiple hypotheses mapped onto a unique, multi‐dimensional search space (Lorenz, Hampshire, and Leech 2017). Although the present study demonstrated that the NBO approach can be successfully applied to this simple one‐dimensional space, it can also be used to explore more complex search spaces that can be more informative of the heterogeneity of the social stimuli infants encounter and select in real life (Gui et al. 2024; Throm et al. 2023). One potential future application could include young children with neurodevelopmental conditions. For example, as described above, applying NBO to studying autistic toddlers’ individual responses to familiar versus unfamiliar faces could reveal insights about individual attentional preferences at that stage. Further, NBO can be used in a live interaction setting, in which different dimensions of the space reflect distinct aspects of social behaviour (e.g., variation in eye gaze; Throm et al. 2023) and further give insight to progressive specialisation on the individual level in other contexts including other modalities, such as social and nonsocial sounds, or other cognitive tasks, such as joint attention.

## 5 Conclusion

The present study applied NBO to infant ERP data and showed that it is possible to obtain reliable measures of infant neural responses reflecting attention engagement on the individual level, achieving lower attrition rates than in traditional studies. Although attentional preferences for parent versus stranger did not show up on a whole‐group level, NBO revealed heterogeneous individual attentional preferences. Indeed, averaging responses across infants with attentional preference for the familiar versus unfamiliar face revealed the effect on the subgroup‐level. This is particularly relevant in developmental and neurodiverse samples where studies averaging across individuals would miss detecting differences (Haartsen, Gui, and Jones 2024). In this study, age and parent‐report measures of social behaviour were not related to attentional preferences. Further analyses with additional measures of social behaviour and the social environment are needed, as well as future studies including a broader age range to investigate a potential age effect beyond the age boundaries applied in the present study. By efficiently mapping an individual's response function to a stimulus space, NBO is a promising new tool to unveil attentional preferences in different subgroups and individuals. By leveraging individualised paradigms that allow children to choose what to attend to such as NBO, researchers will have new tools to include and encourage participation of neurodiverse individuals in neuroimaging research. More generally, NBO embeds real‐time data quality checks and fosters replicability of results by setting analysis parameters before collecting the data, thus providing an experimental design that fulfils core requirements of open science towards more robust and replicable research findings.

## Disclosure

The funders had no role in the design of the study; in the collection, analyses, or interpretation of data; in the writing of the manuscript, or in the decision to publish the results. Any views expressed are those of the author(s) and not necessarily those of the funders.

## Ethics Statement

This study was approved by the Departmental Ethics Committee of the Department of Psychological Sciences, Birkbeck College, University of London. The study conforms to the Declaration of Helsinki. Caregivers gave their informed consent for their child to take part in the study.

## Conflicts of Interest

The authors declare no conflicts of interest.

## Supporting information



Supporting‐Information

## Data Availability

The data that support the findings of this study are available on the UK Data Service repository (doi: 10.5255/UKDA‐SN‐856214).
