# Supplementary material for: Combining Real‐Time Neuroimaging With Machine Learning to Study Attention to Familiar Faces During Infancy: A Proof of Principle Study
Source: Dev Sci. 2024 Nov 26;28(1):e13592. doi: 10.1111/desc.13592 (PMC11599787; doi:10.1111/desc.13592)
Supplement: Supplementary file 1 — Supporting‐Information [file DESC-28-e13592-s001.docx]

**Supporting Information**

**Supporting Information 1***Procedures to monitor and attract the infant’s attention to the screen*

At the start of each block, i.e. before the first trial, a spinning red spiral was presented centrally to attract the infant’s attention to the screen. The experimenter monitored the infant’s gaze through a webcam and elicited the disappearance of the red spiral and the start of the first trial per key press once the infant was looking at the screen. If the infant was looking away during the experiment, the experimenter released the key press and the red spiral re-appeared before the next trial was presented. If needed, a second experimenter intervened to bring the infant’s attention back to the screen. After the 4^th^, 7^th^ and 9^th^ trial, a colourful cartoon video paired with infant-friendly music was presented to further enhance the infant’s attention to the screen and reduce habituation to the faces. The caregiver was asked to not interact with the infant during the stimulus presentation, besides to gently hold their hands if they tried to grab the cables. In the break between two blocks, the infant was having a break from stimulation. If the infant was fussy, the caregiver was asked to naturally interact with them, or an experimenter handed them a teething ring.

**Supporting Information 2**

*Artifact removal*
Automatic artifact detection allowed us to exclude from each channel each trial in which a) the signal exceeded the defined amplitude threshold, b) the signal exceeded the defined range threshold, or c) if the signal was consistently flat (below 0.0001 μV). To take into account the general ERP magnitude of an individual, amplitude and range thresholds for artifact detection were determined for each individual at the beginning of the experiment. To do this, the script calculated the Nc obtained after the first block with more lenient thresholds (amplitude: +/-250 μV; range: of +/-500 μV). If the peak amplitude of this Nc was below +/-200 μV in amplitude, the script used more conservative thresholds for artifact rejection for that infant (amplitude: +/-200 μV; range: of +/-400 μV). In that case, the script re-calculated the ERP from the first block using the adapted thresholds.

**Supporting Information 3**

*Calculation of the Nc mean negativity*First, the point of the lowest amplitude of the signal within the Nc time window was identified as the peak of the Nc. If this peak was negative, the onset (i.e., when the signal first crossed the x-axis before the peak) and offset (i.e., when the signal first crossed the x-axis after the peak) of the negative deflection around this peak was determined. If the onset or offset point was located beyond the boundaries of the classic Nc time window (250 and 800 ms), the respective boundary of the Nc time window was used as onset or offset. If the duration between onset and offset was at least 58 ms, indicating the negative deflection reflected a brain state of attention engagement with faces (Gui, 2019), the mean amplitude of the signal within the defined onset or offset point was calculated and saved as EEG target value. If the duration of the negative deflection was shorter than 58 ms, or the peak was positive, that is no negative deflection was identified, the mean amplitude across the entire Nc time window (250-800 ms) used in traditional experiments, instead of only of the negative deflection, was used as EEG target metric in the respective block.

**Supporting Information 4***Specifics of the Bayesian Optimisation algorithm*To build the surrogate model, we used Gaussian process regression with a Mátern covariance kernel which does not require assumptions about the underlying function. The relation between exploration and exploitation is specified in the hyperparameter ξ of the acquisition function, with a lower ξ favouring exploitative sampling towards rapid identification of the maximum, and a higher ξ favouring exploratory sampling towards extensively mapping the brain function across the stimulus space. In the present study, we set ξ to 0.1, which allowed us to prioritise identification after some exploration of the space. In the present study, *Expected Improvement (EI)* was used as acquisition function, allowing the algorithm to explore the space before then rapidly converging to the predicted optimum (e.g., Brochu, Cora, & de Freitas, 2010).
Four pre-defined images were presented to all infants at the start of the paradigm (“burn-ins”) for the algorithm to create an initial statistical model of the EEG target metric across the space before entering the optimization phase. Burn-in points were the two extremes of the 1-dimensional search space and two images from between, so that the spatial distance between each burn-in image was constant (stranger’s face – ⅓ parent’s face – ⅔ parent’s face – parent’s face). The four burn-in images were presented to all infants in randomly assigned order. A control analysis to ensure that the order of the burn-in images did not affect the optimum revealed that the correlation between the space-position of the first burn-in and the space-side of convergence (familiar vs unfamiliar side) was not significant (*χ2*(3, *N* = 51) = 1.771, *p* = .621).

**Supporting Information 5***Description of the infant behaviour questionnaires*
Parents were asked to fill in online questionnaires before their visit to the lab. The questionnaires used in this study were the Vineland Adaptive Behavior Scale (VABS) and the Infant Behavior Questionnaire-Revised (IBQ-R; Gartstein & Rothbart, 2003). All questionnaires were administered online using the Gorilla online experiment builder (<https://www.gorilla.sc> (Anwyl-Irvine, Massonnié, Flitton, Kirkham, & Evershed, 2020). The database initially planned to be used at the time of the preregistration (RedCap, <https://www.project-redcap.org>) was not used due to administrative reasons.

The Vineland Adaptive Behavior Scales (VABS) is a standardised tool measuring adaptive behaviour in everyday (Sparrow, Cicchetti & Balla, 2005). It has been extensively used to capture variability in adaptive behaviour and differences in developmental trajectories in infants and toddlers (Bussu et al., 2018; Estes et al., 2015). The Infant Behavior Questionnaire-Revised (IBQ-R) is a widely used parent-report measure developed to assess dimensions of temperament in infants between 3 months and one year of age (Gartstein & Rothbart, 2003). This tool allows to reliably measure individual differences in reactivity (e.g., arousability of emotional, motor, and attentional responses) and self-regulation (processes that modulate reactivity, such as attention) that complement laboratory assessments (Bosquet Enlow, White, Hails, Cabrera, & Wright, 2016)

In order to specifically evaluate the relationship between optimum-parent distance and behaviour towards familiar and non-familiar people, we calculated three indices composed of specific questions selected from the VABS or IBQ-R: Interest in familiar persons, interest in unfamiliar persons, distress towards unfamiliar persons. Table S1 lists the items of the VABS that were combined to the index variables Interest in a familiar person and Interest in a new person, respectively, and the items of the IBQ that were combined to the index variable Distress towards other persons.

**Supporting Information 6***Table SI 1.* Indices derived from items of the Vineland Adaptive Behavior Scales (Sparrow, Cicchetti & Balla, 2005) and Infant Behavior Questionnaire (Rothbart, 1981; Gartstein & Rothbart, 2003; Putnam et al., 2014) – revised to measure interest and distress towards familiar and unfamiliar people. *Of note, VABS items cannot be displayed due to copyright restrictions.


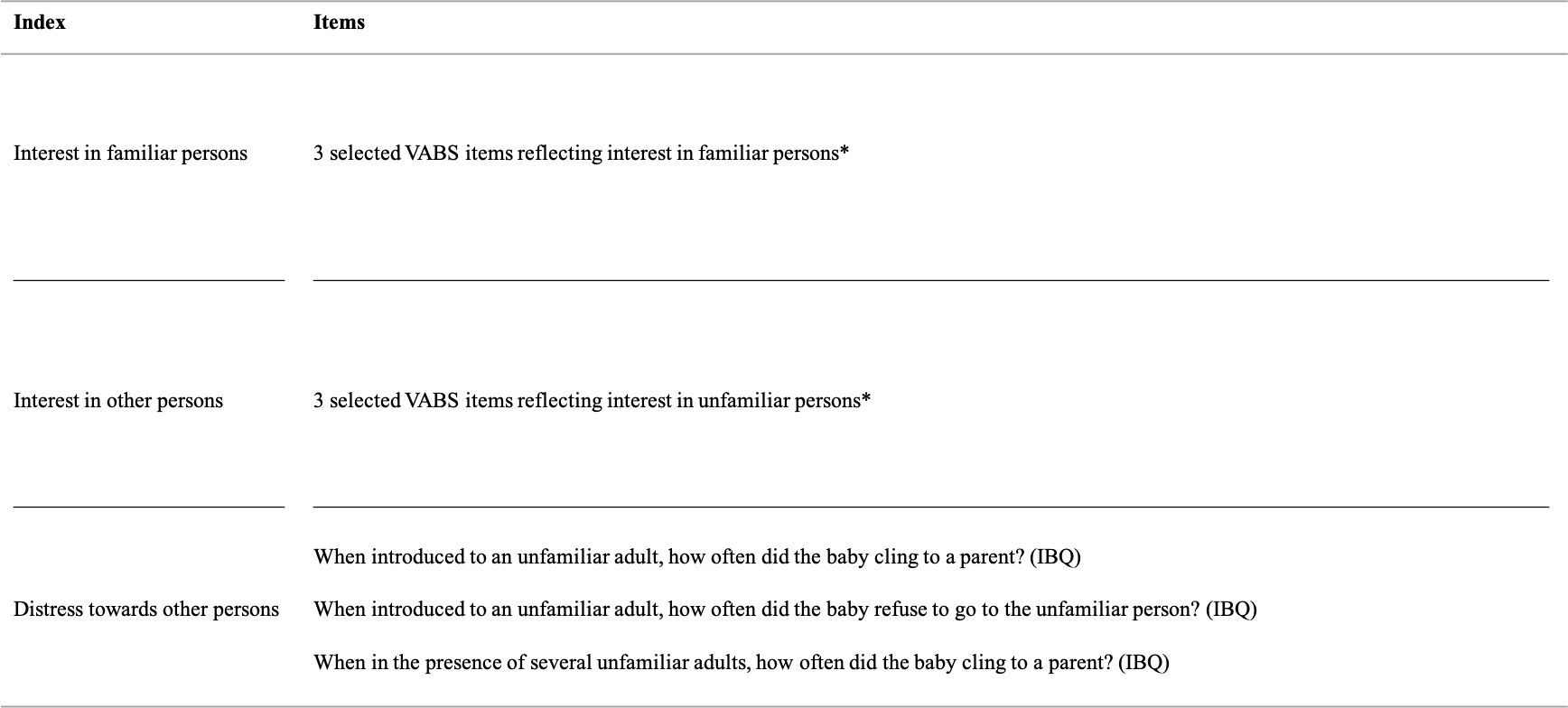


**Supporting Information 7**

*Additional analyses accounting for the dichotomous distribution of the dependent variable*

Because the individual optima were not normally distributed across the 1-dimensional stimulus space but clustered over the two extremes (original parent, original stranger), we conducted additional logistic analyses. As dependent variable in these logistic analyses, instead of the Euclidean distance from parent’s face in the stimulus space, we used the likelihood of converging at either the parent- or the stranger-side of the stimulus space.

For the relation with age, Fisher’s test revealed that the *likelihood of converging for the parent vs stranger* side was not related to age group (*n* = 52, *p* = .402). Further, logistic regression revealed that the *likelihood of converging for the parent vs stranger* side was not related to age in days (*β* = .010, *SE* = .006, *p*=.081, η_p_^2^ = 1.79). Adding in rated parent-stranger similarity as covariate did not change the pattern of results.

For the relation with behaviour, multiple logistic regression revealed that the *likelihood of converging for the parent vs stranger* side was not related to interest in familiar persons (*β* = -.503, *SE* = 1.380, *p*=.716), interest in other persons (*β* = -.034, *SE* = 1.269, *p*=.979) or distress towards other persons (*β* = -.181, *SE* = .297, *p*=.541). Adding in rated parent-stranger similarity as covariate did not change the pattern of results.

**Supporting Information 8**

*
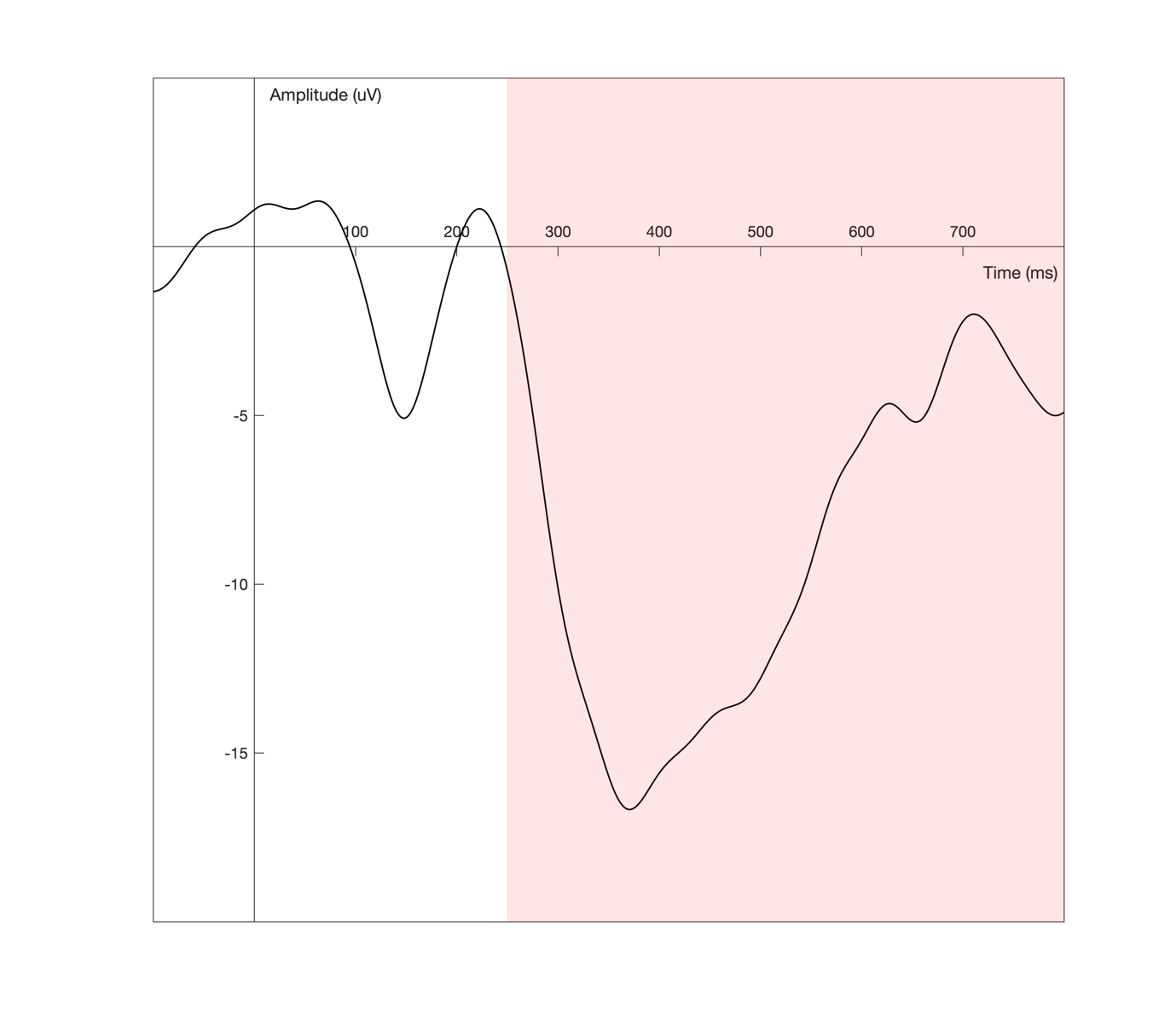
*

*Figure SI 1.* Grand average Nc wave form

**Supporting Information 9**

We computed additional checks to assess whether habituation or repetition suppression have influenced the results. To ensure the Nc negativity did not decrease as a function of block number, we calculated a linear mixed model (R-package “lmerTest”) with Nc negativity as dependent variable, block number as fixed factor and participant as random factor. Block number did not significantly affect the Nc negativity (β = -0.290, SE = 0.192, p=.132). Further, to test whether within an infant the burn-in image presented first was more likely to be identified as optimum than the other burn-in images, we calculated the proportion of infants for whom their optimum corresponded to the first image presented. The Bayesian Optimisation algorithm converged on the first burn-in image for 18.0% of the infants (N=8), which is not more often than expected by chance (25%; 4-sample test for equality of proportions: χ2(3)= 0.85854, p=0.8354). Figure SI 1 shows the Nc mean negativity for each participant (individual black dots) as a function of the number of blocks, indicating that the Nc mean negativity does not systematically decrease as a function of increasing block number.

*
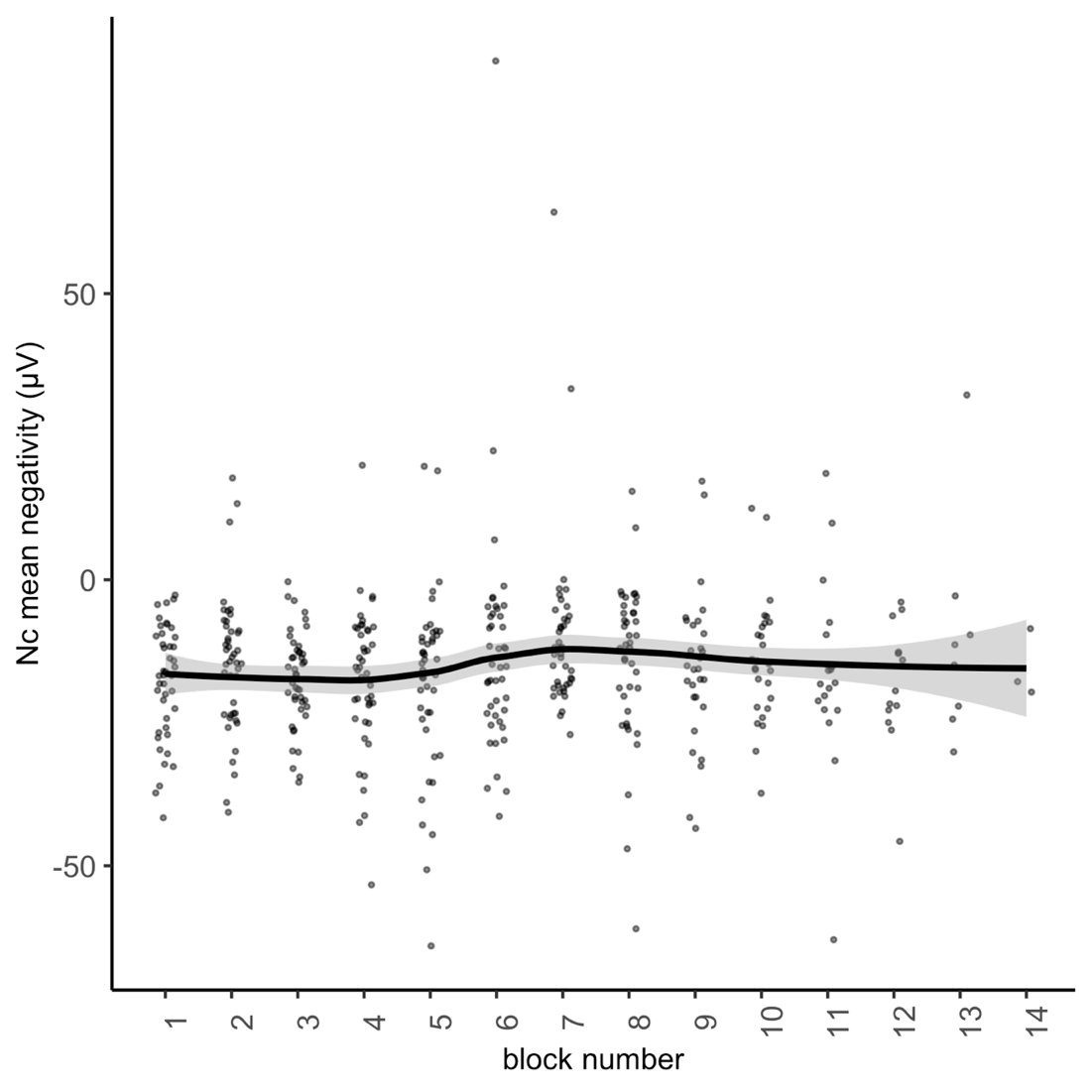
*

*Figure SI 2.* Nc mean negativity as a function of block number.

**References**

Anwyl-Irvine, A. L., Massonnié, J., Flitton, A., Kirkham, N., & Evershed, J. K. (2020). Gorilla in our midst: An online behavioral experiment builder. *Behavior Research Methods*, *52*(1), 388–407. https://doi.org/10.3758/s13428-019-01237-x

Bosquet Enlow, M., White, M. T., Hails, K., Cabrera, I., & Wright, R. J. (2016). The Infant Behavior Questionnaire-Revised: Factor structure in a culturally and sociodemographically diverse sample in the United States. *Infant Behavior and Development*, *43*, 24–35. https://doi.org/10.1016/j.infbeh.2016.04.001

Brochu, E., Cora, V. M., & de Freitas, N. (2010). A Tutorial on Bayesian Optimization of Expensive Cost Functions, with Application to Active User Modeling and Hierarchical Reinforcement Learning. *arXiv,* 1012.2599, <http://arxiv.org/abs/1012.2599>

Bussu, G., Jones, E. J. H., Charman, T., Johnson, M. H., Buitelaar, J. K., Baron-Cohen, S., … Volein, A. (2018). Prediction of Autism at 3 years from behavioural and developmental measures in high-risk infants: A longitudinal cross-domain classifier analysis. *Journal of Autism and Developmental Disorders*, *48*(7), 2418–2433. https://doi.org/10.1007/s10803-018-3509-x

Estes, A. M., Zwaigenbaum, L., Gu, H., St. John, T., Paterson, S., Elison, J. T., … Piven, J. (2015). Behavioral, cognitive, and adaptive development in infants with autism spectrum disorder in the first 2 years of life. *Journal of Neurodevelopmental Disorders*, *7*(1), 1–10. https://doi.org/10.1186/s11689-015-9117-6

Gui, A. (2019). *Evaluating the role of social attention in the causal path to Autism Spectrum Disorder*. <http://bbktheses.da.ulcc.ac.uk/id/eprint/444>

Gartstein, M. A., & Rothbart, M. K. (2003). Studying infant temperament via the Revised Infant Behavior Questionnaire. *Infant Behavior and Development*, 26 (1), 64-86.

Putnam, S. P., Helbig, A. L., Gartstein, M. A., Rothbart, M. K. & Leerkes, E. (2014). Development and Assessment of Short and Very Short Forms of the Infant Behavior Questionnaire-Revised. *Journal of Personality Assessment*, 96, 445-458.

Rothbart, M. K. (1981). Measurement of temperament in infancy. *Child Development*, 52, 569-578.

Sparrow, S. S., Cicchetti, D. V., & Balla, D. A. (2005). Vineland adaptive behavior scales Vineland-II: Survey forms manual. Minneapolis, MN: Pearson.
